# Dentists with enhanced skills (Special Interest) in Endodontics: gatekeepers views in London

**DOI:** 10.1186/s12903-015-0085-8

**Published:** 2015-09-21

**Authors:** Swapnil G. Ghotane, Mustafa Al-Haboubi, Nick Kendall, Claire Robertson, Jennifer E. Gallagher

**Affiliations:** King’s College London Dental Institute at Guy’s, King’s College and St Thomas’s Hospitals, Division of Population and Patient Health, Denmark Hill Campus, Bessemer Road, London, SE5 9RS UK; Faculty of Education & Health, University of Greenwich, Avery Hill Road, London, SE9 2UG UK; NHS South West London and NHS London, based at Public Health Directorate, Floor 13, Leon House, 233 High Street, Croydon, CR9 1XT UK; NHS Westminster, 15 Marylebone Road, London, NW1 5JD UK

**Keywords:** Endodontics, General dental practices, Dentistry, Dentist, Models of care, Oral health policy

## Abstract

**Background:**

Dentists with a special interest hold enhanced skills enabling them to treat cases of intermediate complexity. The aim of this study was to explore primary dental care practitioners’ views of dentists with a special interest (DwSIs) in Endodontics in London, with reference to an educational and service initiative established by (the former) London Deanery in conjunction with the NHS.

**Methods:**

A cross-sectional postal survey of primary care dentists working across different models of care within London was conducted, with a target to achieve views of at least 5 % of London’s dentists. The questionnaire instrument was informed by qualitative research and the dental literature and piloted prior to distribution; data were analysed using SPSS v19 and STATA v12.0.

**Results:**

Six per cent of London’s primary care dentists (*n* = 243) responded to the survey; 53 % were male. Just over one third (37 %; *n* = 90) were aware of the DwSI service being provided. Most practitioners reported that having access to a DwSI in Endodontics would support the care of their patients (89 %; *n* = 215), would carry out more endodontic treatment in the NHS primary dental care if adequately reimbursed (93 %; *n* = 220), and had more time (76 %; *n* = 180). Female respondents appeared to be less confident in doing endodontic treatment (*p* = 0.001). More recently qualified respondents reported greater need for training/support for performing more endodontic treatment in the NHS primary dental care (*p* = 0.001), were more dissatisfied with access to endodontic service in the NHS primary dental care (*p* = 0.007) and more interested to train as a DwSI in endodontics (*p* = 0.001) compared with respondents having a greater number of years of clinical experience since qualification.

**Conclusion:**

The findings lend support to the concept of developing dentists with enhanced skills as well as ensuring additional funding, time and support to facilitate more routine endodontics through the NHS primary care to meet patient needs. More recently qualified dentists working in London were more concerned regarding endodontic service access, expressed need for training/support for undertaking more endodontic treatment in the NHS primary dental care and a desire to train as a DwSI in endodontics.

## Background

Primary dental care practitioners are the main providers of dentistry acting as ‘gatekeepers’ to specialist care within England [1–4]; it is imperative to explore their views on models of care. This paper reports their views on a combined educational and service initiative in London, established by the former London Deanery and the National Health Service (NHS), in conjunction with commissioners from former NHS management organisations (Primary Care Trusts or PCTs).

### Dentistry and endodontic care in the England

Dentistry in England is provided through a blend of public (NHS) and private services [5]. The majority of dental service is provided in primary care, mostly in NHS primary dental care, which operates on a co-payment system, wherein the NHS dentists are remunerated under three bands of ‘units of dental activity (UDAs)’ [6, 7].

All dentists nationally are trained to provide routine endodontics in line with General Dental Council (GDC) requirements for education [8, 9]. Endodontic treatment at primary care level is remunerated under a banded system, each of which has a specific ‘menu’ of care. Band 1 is essentially an oral health assessment; Band 3 a course of care involving laboratory work and Band 2 all other courses of routine care that is not ‘Urgent’ care [7]. Since the introduction of this banded payment system in 2006, the level of endodontic care provided in the primary care sector has reduced [5, 10–12], with increased referrals to hospital services [13].

Specialist services are limited. As of July 2014, there are over 40,000 dentists on the UK dental register which includes 263 specialists in endodontics, and although there are 310 in the over-arching specialty of restorative dentistry, many of the latter do not focus on endodontics or are on both registers [14].

### Development of the concept of Dentists with Special Interest (DwSI)

The emphasis nationally is on developing innovative models of care and local workforce education and training to meet local need [15, 16]. The past decade has seen a move to shift specialist services into primary care settings with a view to improving NHS health services in terms of access, quality care and cost-effectiveness [17–21]. This led to the concept of dentists with special interest (DwSIs), following similar medical initiatives [22, 23]. The DwSI concept advocated training general dentists who would be working in primary care and provide enhanced services in addition to those in their generalist role [22]. They would act as independent practitioners working within the limits of their competence, referring patients to secondary (hospital) care whenever necessary and operating within NHS clinical networks. The terminology has evolved during the course of this research and the terms 'extended skills' and 'enhanced skills' are increasingly used rather than 'special interest'.

### DwSI in endodontics

The importance of providing advanced dental services was highlighted in a review of English NHS Dental Services in 2009 [24], with the expectation that such services may utilise general dentists with enhanced skills. Williams *et al.* (2010) highlight the importance of carefully planning any shift to out-of-hospital services and understanding the effectiveness of any alternate care pathway on the local population to form an informed future action [21]. In response to the English review, and patient needs, a two-year pilot programme to train dentists with special interest (DwSIs) in endodontics was commissioned by the former London Deanery in conjunction with the former London PCTs. The Postgraduate Dental Dean of the former London Deanery cited the aim of this programme as “*to reduce unnecessary referrals to hospitals, reduce extraction rates and train GDPs to undertake complex endodontic procedures within the Primary Care sector*” [25].

Out of the nine dentists (trainee DwSIs) successful in being selected across eight different London PCTs to undertake the training programme, eight completed the training, with one discontinuing after the mid-course evaluation. The host PCT of the trainee DwSI funded the service component of this scheme with the aim of developing their endodontic capacity in the short-term and building capacity for the future.

It is important to establish the views of primary dental care practitioners on new initiatives as they are the ‘gatekeepers’ of patient access to DwSI and specialist services. The success of such a programme (designed as a resource for primary care practitioners, and to strengthen and expand the clinical networks operating within the NHS) depends on its utilisation by these professionals. Evaluation of new initiatives is important in shaping the delivery of health care through evidence [21].

### Aim

The aim of the study was to explore primary dental care practitioners’ views of dentists with a special interest (DwSIs) in Endodontics in London, and how these views varied by sex, length of time since qualification, possession of postgraduate qualifications and awareness of the DwSI scheme. The objectives of this study were to:

1. Assess primary dental care practitioners’ awareness of endodontic DwSI services in London.
2. Assess acceptability of endodontic DwSI services to primary dental care practitioners.
3. Assess perceived needs for and advantages/disadvantages of endodontic DwSI services.
4. Measure the interest amongst primary dental care practitioners to provide endodontics and/or train as a DwSI.
5. Determine the views on the most appropriate pathway of referring patients to DwSI services.

Since the conclusion of this research, the NHS has undergone radical reorganisation of administrative structures [26]. The responsibilities of London Deanery have been transferred to Health Education England (NW London) and NHS England has become responsible for commissioning all dental services as part of a national system [27]. Furthermore, there is a greater focus on ‘dentists with enhanced skills’ (DES), rather than DwSIs; however, as primary dental care practitioners were questioned about the concept under the label ‘DwSIs’, this terminology has been used in reporting this research; although, the implications are discussed in relation to the evolving policy emphasis on ‘dentists with enhanced skills’(DES).

## Methods

A questionnaire instrument was derived through published literature and qualitative research, using Dillman's approach to surveys [28]. The qualitative aspects of the wider study [29] were conducted in the form of semi structured interviews using a topic guide and involved stakeholders from Health Education England (former London Deanery), course participants, dental public health consultants, trainers and educators, commissioners from the clinical commissioning groups (formerly PCTs), specialists and general dental practitioners involved with the pilot. The findings of the qualitative research informed the content of the questionnaire. The questionnaire consisted of a combination of 31 closed and open ended questions exploring primary dental care practitioners’ views regarding the DwSI services in endodontics, their awareness and use of the pilot service, changes required to endodontics services and their personal interests in enhancing the skills. It was piloted on primary dental care practitioners, modified in light of feedback and re-tested on another sample of practitioners to improve the phrasing of questions, before being distributed by post. Approval was provided by King’s College London Research Ethics Committee (Ref-BDM/11/12-24).

There are around four thousand practitioners across one thousand primary dental practices in London [30]. A 5 % sample of dentists in London (circa 211) was sought for this pilot project. The sample size calculation was based on the ‘proportion test’, i.e. testing the proportion of dentists who consider a DwSI service useful for patients. It was assumed that for any new service to be termed as useful, at least three quarters (75 %) of the sample should be in favour. Therefore, a study with 80 % power and an effect size of 0.08 will require at least a sample of 211 to detect a significant difference between the sample and the population proportions of 0.75 at the 5 % level of significance. Based on the response rate of 52 % from similar research with GDPs in inner south east London [31], and anticipating a low response, 799 dentists across six selected boroughs of London were invited to participate in a survey and thus, capture the views of practitioners working in a range of different contexts. Questionnaires were therefore sent to all practitioners providing NHS dentistry within six boroughs which corresponded with representative PCTs taking account of the following criteria:

- Presence of DwSI, or not
- Presence of dental teaching hospital/hospital providing specialist restorative dental services in the PCT area, or not
- Levels of routine NHS endodontic activity in primary dental care in the previous year
- Whether the DwSI was accepting external referrals, and the nature of patient triage
- Inner/outer London locations
- Type of service: salaried or general dental practice

Recruitment was conducted at practitioner level. The list of eligible practitioners and their postal addresses was obtained from the PCTs involved in the study and checked against internet sources. In accordance with the Dillman’s protocol for postal surveys [32], researchers made contact with the London primary dental care practitioners on up to five separate occasions over the course of eight weeks in early 2011. The practitioners did not receive any incentive and were under no obligation to complete the questionnaire. They were informed that their completion of the questionnaire would imply their consent and subsequent publication of the data for this study.

## Data analysis

Participant responses were entered onto a computer and analysed using the statistical package SPSS v19.0 and STATA version 12.0. Respondents were asked to rate whether or not they agreed with a series of statements regarding the provision of NHS and DwSIs in endodontics service and these statements were grouped into five different categories according to their relevance. The five categories were namely ‘Confidence to undertake endodontic treatment’, ‘Training/Support for undertaking more endodontic treatment in NHS primary dental care’, ‘Dissatisfaction with endodontic service access’, ‘Acceptability/Support for DwSI services’ and ‘Interest in training as an endodontic DwSI’. ‘Confidence to undertake endodontic treatment’ consisted of items exploring the confidence of respondents in carrying out endodontic treatment. ‘Training/Support for undertaking more endodontic treatment in NHS primary dental care’ consisted of statements which explored whether providing training or specialist support for respondents would result in them carrying out more endodontic treatment in primary dental care. The third category ‘Dissatisfaction with endodontic service access’ had items related to dissatisfaction with access to endodontic services in NHS primary dental care whereas ‘Acceptability/Support for DwSI services’ consisted of statements on acceptability and support for DwSI services in endodontics. And finally, ‘Interest in training as an endodontic DwSI’ outcome explored respondents’ desire to train as a DwSI in endodontics.

Descriptive statistics were used to summarise the findings. The five different categories were compared by sex using independent samples *t*-test given differences in motivation and career expectations [33–35]. Multivariate regression analysis was used to identify the significant predictors for the different categories. The model included predictor variables including demographics such as sex, respondent’s number of years since qualification (BDS), respondent’s awareness of DwSI scheme and respondents having a post-graduate qualification as the predictor variables and the category scores as the outcome measure. Logistic regression was carried out to test the effect of predictors on the referral of patients to DwSI service in endodontics. Independent samples t- test was carried out to compare the mean number of years since qualification between respondents who were interested and not interested to train as a DwSIs in Endodontics. Statistical significance was considered at p ≤ 0.05. Thematic analysis of responses to open questions was undertaken [36].

## Results

### Survey response and demographics

Of the 799 dentists with valid addresses invited to take part in the survey, 243 (30 %) returned a completed questionnaire, representing almost six percent of primary care dentists in London. Ninety one questionnaires were returned by the postal service because the dentist address was not valid and ten questionnaires were returned by the participants who considered themselves ineligible. The response rate by PCT varied from 18 % to 47 %. Males constituted just over half (53 %) of the respondents. Five percent reported having a post-graduate qualification in endodontics. The clinical experience of the respondents ranged from one to 51 years (Mea*n* = 16 years). Practices ranged from one to 10 surgeries in size, with the mode being two (31 %). Eighty percent of respondents worked in just one practice within the PCT, whereas the remainder covered two or three practices.

### Univariate and multivariate analysis of respondents’ views

Table 1 summarises the results of univariate analysis of different categories for comparison between male and female respondents. Female respondents were less confident in undertaking endodontic treatments (*p* = 0.001) and more supportive of the DwSI services as compared to male respondents (*p* = 0.047).

**Table 1:** Results of univariate analysis for comparing categories by sex of respondent (*n* = 237)

Category	Male (*n* = 129)	Female (*n* = 108)	*P*-value
	Mean (sd)	Mean (sd)	
Confidence to undertake endodontic treatment	11.34 (2.65)	09.87 (2.83)	0.001*
Training/Support for undertaking more endodontic treatment in NHS primary dental care	11.33 (2.48)	11.12 (2.41)	0.51
Dissatisfaction with endodontic service access	11.72 (2.25)	12.05 (2.19)	0.051
Acceptability/Support for DwSI services	15.09 (2.05)	15.58 (1.68)	0.047
Interest in training as an endodontic DwSI	3.48 (1.36)	3.51 (1.41)	0.82

Note: *Significance at 1 % level (p<0.01)

Multivariate regression analysis for five categories is presented in Table 2. ‘Confidence’ in undertaking endodontic treatment was significantly lower in female respondents (*p* = 0.001) and those unaware of the DwSI service (*p* = 0.02); whereas confidence was higher in respondents having any post graduate qualification (*p* = 0.04), as might be expected. In relation to ‘training/support for undertaking more endodontic treatment in the NHS primary dental care’, female respondents (*p* = 0.04) and those with more years of experience post-qualification (*p* = 0.001) were less likely to undertake more endodontics treatment, even if they had more training or specialist support for endodontics. ‘Dissatisfaction with endodontic service access’, was more common in respondents’ who had qualified more recently compared with their those had qualified longer (*p* = 0.007). In relation to ‘Interest in training as an endodontic DwSI, respondents more recently qualified were significantly more interested in training as a DwSI in endodontics (*p* = 0.001). There was no difference across groups in relation to their ‘acceptability/support for DwSI services’.

**Table 2:** Results of multivariate linear regression analysis for the five key categories

Category	Reference	Effect	95 % Confidence Interval		*P*-value
			Lower Confidence Limit	Upper Confidence Limit	
Confidence to undertake endodontic treatment					
Female	Male	−1.57	−2.32	−0.83	0.001*
Years since qualification	^a^	−0.03	−0.06	−0.06	0.14
Not aware of DwSI service	Aware of DwSI service	−0.84	−1.57	−0.12	0.02**
Not having Endo PG qualification	Having Endo PG qualification	−0.31	−1.93	1.32	0.71
Having other PG qualification	Having Endo PG qualification	2.38	0.08	4.69	0.04**
Training/Support for undertaking more endodontic treatment in NHS primary dental care					
Female	Male	−0.70	−1.37	−0.03	0.04**
Years since qualification	^a^	−0.06	−0.09	−0.03	0.001
Not aware of DwSI service	Aware of DwSI service	0.05	−0.60	0.70	0.89
Not having Endo PG qualification	Having Endo PG qualification	0.40	−1.06	1.86	0.59
Having other PG qualification	Having Endo PG qualification	1.09	−0.98	3.15	0.30
Dissatisfaction with endodontic service access					
Female	Male	−0.02	−0.63	0.59	0.94
Years since qualification	^a^	−0.04	−0.07	−0.01	0.007*
Not aware of DwSI service	Aware of DwSI service	0.48	−0.12	1.07	0.12
Not having Endo PG qualification	Having Endo PG qualification	−0.94	−2.27	0.39	0.17
Having other PG qualification	Having Endo PG qualification	−1.43	−3.32	0.46	0.14
Acceptability/Support for DwSI services					
Female	Male	0.37	−0.16	0.90	0.17
Years since qualification	^a^	−0.02	−0.04	0.01	0.15
Not aware of DwSI service	Aware of DwSI service	−0.14	−0.65	0.38	0.60
Not having Endo PG qualification	Having Endo PG qualification	−0.18	−1.33	0.97	0.76
Having other PG qualification	Having Endo PG qualification	−0.44	−2.07	1.20	0.60
Interest in training as an endodontic DwSI					
Female	Male	−0.30	−0.67	0.07	0.11
Years since qualification	^a^	−0.04	−0.06	−0.02	0.001*
Not aware of DwSI service	Aware of DwSI service	0.11	−0.25	0.48	0.54
Not having Endo PG qualification	Having Endo PG qualification	0.15	−0.67	0.96	0.72
Having other PG qualification	Having Endo PG qualification	0.85	−0.30	2.00	0.15

Note: ^a^was a continuous variable. Higher scores indicate more confidence/support/dissatisfaction/acceptability/interest

Abbr. - *PG*: Post graduate; *DwSI*: Dentist with Special Interest; *Endo*: Endodontic

Note: *Significance at 1 % level (p<0.01)

**Significance at 5 % level (p<0.05)

### Awareness of DwSI in endodontics scheme

Thirty seven percent (*n* = 90) of respondents were aware of the DwSI training scheme; however, there was a significant difference in the level of awareness between dentists in the PCTs which had a DwSI scheme compared with those where there was none, or the trainee was only accepting internal practice referrals (78 % cf 11 %; *p* = 0.01). In PCTs that had DwSI schemes, 54 % (*n* = 59) of respondents had referred patients to the services, 82 % (*n* = 49) of which were to other dental practices. The most common reasons for non-referral (obtained through open questions) were ‘not being aware of the service’ and 'preferring to treat patients themselves'. Logistic regression analysis was carried out to test the effect of predictors on the referral of patients to DwSI service (Table 3). Unsurprisingly, the odds of referring patients to DwSI in endodontics scheme was significantly lower (OR = 0.23; *p* = 0.01) among the respondents who were not aware of the scheme. Of the respondents who had referred patients to a DwSI scheme, 55 % (*n* = 60) reported that the service was successful, and this was qualified with comments regarding the systems and outcomes as explained in the following section.

**Table 3:** The results of logistic regression analysis for predicting ‘referral’ of patients to DwSI services’

Outcome	Reference	Odds ratio	95 % Confidence Interval		*P*-value
			Upper Confidence Limit	Lower Confidence Limit	
Have you referred patients to DwSI service (*n* = 104)					
Female	Male	0.95	0.39	2.29	0.91
Years since qualification	^a^	0.98	0.94	1.01	0.19
Not aware of DwSI service	Aware of DwSI service	0.23	0.07	0.72	0.01*
Not having Endo PG qualification	Having Endo PG qualification	0.76	0.11	5.11	0.78
Having other PG qualification	Having Endo PG qualification	1	-	-	-

Note: ^a^was a continuous variable

Note: *Significance at 1 % level (p<0.01)

### Perceived advantages of the DwSI service, and areas of concern

**Fig. 1:**
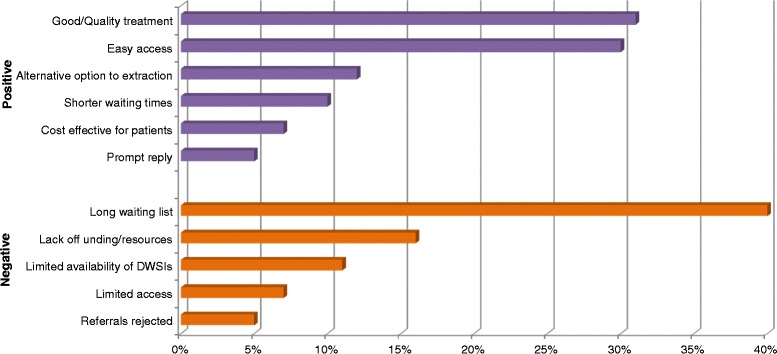
Views of London dentists who have utilized the DwSI service (*n* = 61). This figure gives the proportion of dentists’ views on perceived advantages and disadvantages of DwSI service in London. The dentists responded to an open question and their responses were grouped together for similar themes

Eighty nine percent of respondents (*n* = 215) reported that having access to the services of a DwSI in endodontics would support the care of their patients; 88 % (*n* = 210) felt that DwSIs should accept referrals from outside their practices and 82 % (*n* = 198) reported that they would feel comfortable in referring their patients to a DwSI for treatment. The perceived advantages reported in free text responses to open questions were grouped into ‘better service’ or ‘quality treatment’ for the patients, ‘easy access’, and ‘an alternative option to extraction’ categories (Fig. 1) and demonstrated by following quotes:

“It increases supportive care of my patient and more access to some treatments I am unable to provide”

“It gives patients another option for 'saving' their teeth and may help ease the pressure from hospital services”

Concerns about current DwSI capacity to respond to need were raised by 23 % (*n* = 57) respondents, in which long waiting lists for endodontic care were the major disadvantage (40 %), followed by lack of resources (16 %) and limited availability of the DwSIs (11 %), as illustrated by the following quotations:

“Long waiting list and not enough dentists with DwSI training”

“Long waiting lists-gradually building up …” (*as DwSI became overwhelmed*)

There was a divergence of views on whether the DwSI service might result in loss of patients for general dental practitioners with almost one third supporting and one third opposed to this view and one third equivocal. Similarly, respondents’ views were divided on the issue of whether the DwSI service was needed if the hospitals increased their capacity with almost 35 % (*n* = 84) of dentists agreeing as well as disagreeing with this statement and 31 % equivocal.

### Respondents’ views on referral routes, guidelines and ways to improve, for the DwSI service

**Fig. 2:**
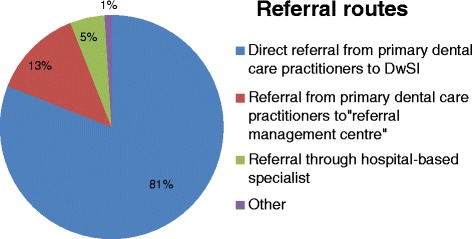
Care pathway for referring patients to the DwSI practitioners (*n* = 242). This figure gives the proportion of London dentists preferring the referral routes to DwSI practitioners

There was a clear message regarding referral routes as 81 % (*n* = 195) of the respondents’ stated 'direct referral to the DwSI' as being their preferred option (Fig. 2). There was diversity of view on the four page-long guidelines for referral to this service. Only half of the respondents (51 %; *n* = 123) perceived them as clear, the remainder considered that it complicated the referral process (20 %; *n* = 48) or set the bar for referral too high (16 %; *n* = 39).

### Respondents’ views on primary and secondary endodontic services

There was a notably high level of dissatisfaction with the present provision of endodontic services in primary and secondary NHS services, with 93 % (*n* = 220) of the respondents reporting that practitioners would carry out more endodontic treatment in the NHS primary dental care if they were reimbursed adequately, or had more time (76 %; *n* = 180). This was illustrated by the following quotations:

"There needs to be better remuneration for RCTs (root canal treatments) on the NHS”

“PCT does not provide enough time-money to perform proper endodontic treatment on the NHS (Band 2 NHS)”

Access to more specialised endodontics was commonly reported as being a challenge; 88 % (*n* = 208) indicated that practitioners are often dissatisfied as a result of difficulties in accessing more specialised services, and 77 % (*n* = 182) felt that these difficulties were a cause for patient dissatisfaction.

Eighty three percent of the dentists referred patients to one or more hospitals in London with 24 % using three or more hospitals. Reasons for not using hospitals included ‘referrals getting rejected’ (15 %); and ‘performing RCT treatment in their own clinics’ (15 %), followed by ‘long waiting list’ (12 %). The main advantage of hospital services was perceived as the provision of high quality treatment to the patients (*n* = 41; 32 %).

### Interest in training as a DwSI

**Fig. 3:**
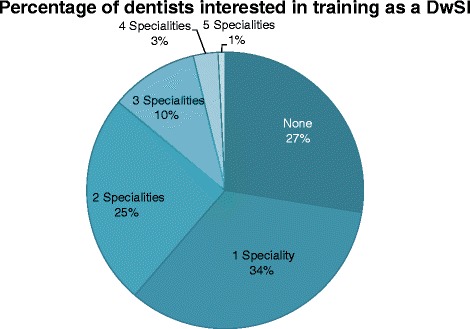
Interest of dentists in training as a DwSI in any branch of dentistry (*n* = 243). This figure gives the proportion of London dentists who are interested to train as a DwSI in any speciality of dentistry. The respondents could tick one or more branches of dentistry

There was support for initiatives to develop DwSIs, with 73 % of the respondents expressing an interest in undertaking DwSI training in one or more branches of dentistry (Fig. 3). Fifty seven percent were personally interested in training as DwSIs in Endodontics, followed by Oral Surgery (40 %), Periodontics and Sedation (both 24 % respectively) and Paedodontics (19 %). A significant difference (*p* = 0.001) in the mean number of years since qualification was found between respondents who were interested and not interested to train as a DwSIs in Endodontics (13.6 years (sd = 12.1) and 19.2 years (sd = 9.1) respectively).

### Estimated need for DwSI service

Looking to the future, 87 % (*n* = 212) of these primary dental care practitioners who responded to this question, estimated that they would refer an average of 24 patients per dentist (range 0-400) per year to DwSIs. Thus, when scaled up to cover the majority of London practitioners, this could translate in an estimated 83,520 cases annually for London (Table 4).

**Table 4:** Response by Borough on Need for DwSI service

Borough	Resident Population (Thousand) [Figures from Mid-2010]	Valid Sample (Dentist)	No. of Dentists who responded	% responded by PCT dentist population	Estimated Number of patients					
					Currently referred to a DwSIs/year			Future referrals to DwSIs/year		
					No. of Dentists who responded	Total No. of Patients	Mean per dentist	No. of Dentists who responded	Total No. of Patients	Mean per dentist
A - no DwSI service, but had secondary endodontic services	287	93	44	47 %	3	6	2	37	1220	33
B - PCT-based triage set-up for the DwSI	229	132	60	45 %	36	670	18.6	52	1340	28
C - DwSI trainee was accepting external referrals	290	181	56	31 %	11	43	4	52	911	17
D - DwSI trainee was in salaried dental services	237	109	26	24 %	1	10	10	24	722	30
E - DwSI trainees accepting only internal referrals	170	91	22	24 %	7	41	6	19	339	18
F - had neither a DwSI trainee nor specialist services	271	193	35	18 %	1	50	50	28	678	24
London	7800	799	243	30 %	59	820	14	212	5210	24

Only 29 % (*n* = 71) identified that they would reduce referral to hospitals if there were DwSIs available with an average of 18 referrals per dentist and a range of 1-200 patients.

## Discussion

### Representativeness of sample population

The survey involved a range of respondents in terms of their clinical experience and practice setup. The demographic characteristics of the study population revealed that the proportion of male and female dentists was similar to that of London and England [30, 37]. Although the response rate was low, the survey superseded its target and captured views of almost six percent of primary care dentists in London following a robust approach [32].

Possible response bias may include respondents having a higher than average interest in post-graduate studies and in becoming a DwSI (range 19 - 57 %), particularly in endodontics (57 %); a higher than expected percentage of respondents reported having a postgraduate qualification in endodontics (5 %). Given the fact there was a considerable interest in becoming a DwSI, this could be an indication of this view; however, interestingly only around half (51 %) of the respondents felt that they enjoyed performing endodontic treatment, thus there was clearly a spread of perspectives amongst those who chose to respond. Although the views may not be representative of primary dental care in general, they represent an important contribution to the debate on dentists with enhanced skills.

### Confidence in endodontic skills

The survey suggested that female respondents were less confident and less likely to provide additional endodontic treatment with further training or specialist support. This approach of female respondents towards endodontic treatment was not affected by other variables such as having a post graduate qualification or number of years since qualification. Females were equally interested to gain enhanced skills training as a DwSI in endodontics as compared with male respondents.

Similarly, respondents who had qualified longer ago reported being less likely to provide additional endodontic treatment with further training/specialist support, but also being less concerned about access to NHS endodontic services. This could be explained by older respondents considering themselves self-sufficient in delivering endodontic treatment and/or accessing endodontic services when required by their patients. In contrast, respondents who had qualified more recently expressed a desire for additional training/support in delivering more endodontic treatment in the NHS primary dental care. This could be attributed to the fact that graduates are now considered ‘safe beginners’ [38] and recognise their need for additional training and support [39]. Additionally, they may still be having more trouble in accessing endodontic services as suggested by their higher levels of dissatisfaction.

Given the higher level of interest in enhanced skills training to become a DwSI amongst more recently qualified respondents, it is possible that they might believe training as a DwSI in endodontics will help them in delivering endodontic treatment to patients, or they might perceive it as an opportunity for career progression. In any case, their interest to further enhance their skills could be harnessed in support of addressing the gap in provision of endodontic service in the NHS primary dental care. Moreover, respondents who had some post graduate qualification appeared to be confident in delivering endodontic treatment. Even if the respondents were not representative of all practitioners, this survey identified 138 London dentists interested in receiving enhanced skills training; this represents a great opportunity to build capacity and address the needs of patients identified in this survey.

### Awareness regarding the pilot DwSI scheme

The survey findings suggested that less than half of the respondents were aware of the scheme. This can be explained by the limited coverage of the educational and service initiative in the region, some PCTs having in-house services and the sampling frame for this survey purposively including both participating and non-participating PCTs. Whilst the findings suggest that awareness of the educational and service initiative was higher in participating PCTs, it was not universal as would be expected with a service that was restricted to participating boroughs (PCTs). This highlights the challenges of promoting new schemes and may also be a reflection of the high level of turnover of dental staff [37]. The study by Pau *et al.* (2010) involving minor oral surgery [40], highlighted the importance of having a communications strategy to endorse the positive finding of such new schemes which would encourage the practitioners to utilise this service for the benefit of their patients. Clear communication on referral pathways within the NHS is very important for the future, particularly if the range of providers increases and there is a regional service.

### Perceived need for and advantages of endodontic DwSI services

The difficulties in accessing hospital endodontic services were highlighted by the findings of this study, together with the potential to reduce referrals and address the gap in service provision by a number of routes within a contemporary care pathway lending support for an established service, ideally in a phased manner with close monitoring and evaluation to ensure that patients are seen in the most appropriate setting. The cost-effectiveness of such schemes, has been suggested in previous studies which evaluated specialist services for Minor Oral Surgery provided in primary care, and reported the potential to reduce the secondary care costs [40–42]; however, moving any endodontic services to primary care DwSIs is unlikely to result in major savings as hospital services are provided on an outpatient tariff, usually within block contracts and dental monies are difficult to extract from ‘block’ contractual arrangements. Second, because there is limited hospital capacity [29]. And third, essentially there is a need for overall service expansion not just a shift in services across settings as additional revenue costs will be incurred. Furthermore, patients currently accessing private dental care may potentially transfer to the NHS sector and put additional demands on the system.

The next clear message emerging from practitioners was the need for change to the present NHS system with regard to the provision of endodontic services; a number of issues raised by the respondents were supported by the wider literature and NHS data. First, practitioners (93 %) felt they were not reimbursed sufficiently for the provision of endodontic services in the NHS primary dental care [6]. Second, there was a view (63 %) that the requirement for certain single use instruments makes the process economically unviable [43]. Third, practitioners (76 %) do not consider they have time to perform endodontic procedures in the NHS primary dental care. Fourth, both practitioners and their patients were dissatisfied as a result of the long waiting lists and by rejected referrals to secondary level services [44]. Fifth, there was evidence that patients who could benefit from endodontic services may be receiving extractions as an alternative treatment because of the gap in service provision [24]. These factors cannot be ignored, and whilst there may be wide support for innovation, all steps should be taken to harness, and use, the skills and training of the generalist professional population, particularly as we move towards dental contract reform [45].

### DwSIs - an innovative model of care using dentists with enhanced skills

The respondents to the survey demonstrated a clear view in favour of the initiative including agreement with the statement that DwSIs in endodontics would support the care of their patients, and support for increasing the number of DwSIs in endodontics. Similar positive views for the DwSIs have been expressed by practitioners across ‘Minor Oral Surgery’ and ‘Periodontics’ [40, 41, 46], which suggests a fundamental shift in the provision of dental care towards developing ‘special interests’ and specialisation in primary care [17–21]. What was striking from the responses was the lack of opposition towards this service.

The majority of respondents (82 %) stated that they would feel comfortable in referring their patients to a DwSI in Endodontics. One quarter of the respondents had referred over 800 patients to DwSI endodontic service. There was a strong feeling amongst respondents that DwSIs should accept referrals from outside their practices (external referrals), and the majority favoured direct referrals to the service, despite there being a concern amongst a minority about losing patients to another dental practice. None-the-less it is clear that some dentists are adapting to new models of working across the health system with cross-referrals between practices. The findings from this, and previous studies [40, 46], underline the potential to increase skills and capacity of the state-funded dental service using both established and innovate models of care [47], which would help in meeting patient needs and facilitate better access to dental care for patients requiring endodontics of moderate complexity. The reviews by Williams *et al.* [21], and Richards [48], suggest changes in care settings and skill-mix may lead to improved access as well as patient and professional satisfaction; hence, the DwSI in endodontics service could aid in the government’s vision of shifting some secondary healthcare further into the community [19, 21]. Furthermore, if the level of need for dentists with enhanced skills is compared with the level of interest in gaining those skills outlined before, it may address the reported need for referrals and subsequently may help in decreasing the burden on the secondary services. These changes would need to be managed in conjunction with service redesign of patient pathways to ensure that as much endodontics is provided in primary dental care as possible, which clearly requires appropriate remuneration.

### Implications for action

There are several implications for policy and strategic action. First, as already highlighted [49], there are implications for the Department of Health in dental contract reforms and NHS England regarding commissioning of primary care dentistry, in that there needs to be a review of the remuneration of endodontic treatment under any new NHS funding arrangements.

Second, the single operating model of dental commissioning and contracting developing through NHS England provides an opportunity to develop skill-mix in the form of dentists with enhanced skills through managed clinical networks in a single operating framework. Innovations such as delivered by this scheme [49] should be formalised within the healthcare system, closely evaluated and shared nationally [27, 50]. This should involve close working of HEE and Public Health England to ensure that the development of dental workforce skills relates to the population health needs.

Third, these findings suggest that there may be significant unmet need for endodontics within the population and this should be addressed in line with national recommendations for primary dental care redesign [24].

Fourth, if schemes of this nature are commissioned in future, there should be adequate measures to ensure appropriate access through agreed criteria for referral so that the services do not become inundated, creating unacceptable waiting lists and dissatisfaction for patients and referring practitioners.

Finally, if the initiative to develop ‘dentists with enhanced skills’ through the work of both dental faculties of the Royal College of Surgeons of England is successful, this will provide an effective means of formalising individuals with these skills within the professional hierarchy [51]; skills that may then be commissioned within the health service.

## Conclusion

The findings of this survey highlight the need for developing an endodontic care pathway to meet patient needs through enhancing the delivery of care by existing primary care services, providing support for developing dentists with enhanced (special interest) skills in endodontics, with services available on direct referral from primary dental care. There was significantly greater interest amongst more recently qualified general practitioners in obtaining additional training and support for primary care provision and developing these skills, which could be harnessed to address the unmet need. The findings suggest there is strong support for this scheme to develop an ‘intermediate’ care level between the primary and secondary services, provided financial and organisational changes are incorporated in the commissioning of endodontics, through patient care pathway development.

## Abbreviations

BDS: Bachelor of Dental Surgery
DES: Dentist with Enhanced Skills
DwSI: Dentist with Special Interest
GDC: General Dental Council
NHS: National Health Service
PCT: Primary Care Trust
RCT: Root Canal Treatment
UDA: Unit of dental activity
